# SPECT Imaging of SST2-Expressing Tumors with ^99m^Tc-Based Somatostatin Receptor Antagonists: The Role of Tetraamine, HYNIC, and Spacers

**DOI:** 10.3390/ph14040300

**Published:** 2021-03-28

**Authors:** Raghuvir Haridas Gaonkar, Fabius Wiesmann, Luigi Del Pozzo, Lisa McDougall, Sandra Zanger, Renata Mikołajczak, Rosalba Mansi, Melpomeni Fani

**Affiliations:** 1 Division of Radiopharmaceutical Chemistry, Clinic of Radiology and Nuclear Medicine, University Hospital Basel, 4031 Basel, Switzerland; raghuvirharidas.gaonkar@usb.ch (R.H.G.); fabiuswiesmann94@gmail.com (F.W.); Luigi.DelPozzo@usb.ch (L.D.P.); Lisa.McDougall@usb.ch (L.M.); Sandra.Zanger@usb.ch (S.Z.); rosalba.mansi@usb.ch (R.M.); 2 Radioisotope Centre POLATOM, National Centre for Nuclear Research, 05-400 Otwock, Poland; renata.mikolajczak@polatom.pl

**Keywords:** ^99m^Tc, SPECT/CT, HYNIC, N4, aminohexanoic acid, spacer, somatostatin receptor antagonists, neuroendocrine tumors

## Abstract

[^99m^Tc]Tc-HYNIC-TOC is the most widely used ^99m^Tc-labeled somatostatin receptor (SST) agonist for the SPECT imaging of SST-expressing tumors, such as neuroendocrine tumors. Recently, radiolabeled SST antagonists have shown improved diagnostic efficacy over agonists. ^99m^Tc-labeled SST antagonists are lacking in clinical practice. Surprisingly, when [^99m^Tc]Tc-HYNIC was conjugated to the SST2 antagonist SS01, SST2 imaging was not feasible. This was not the case when [^99m^Tc]Tc-N4 was conjugated to SS01. Here, we assessed the introduction of different spacers (X: β-Ala, Ahx, Aun and PEG_4_) among HYNIC and SS01 with the aim of restoring the affinity of HYNIC conjugates. In addition, we used the alternative antagonist JR11 for determining the suitability of HYNIC with ^99m^Tc-labeled SST2 antagonists. We performed a head-to-head comparison of the N4 conjugates of SS01 and JR11. [^99m^Tc]Tc-HYNIC-TOC was used as a reference, and HEK-SST2 cells were used for in vitro and in vivo evaluation. EDDA was used as a co-ligand for all [^99m^Tc]Tc-HYNIC conjugates. The introduction of Ahx restored, to a great extent, the SST2-mediated cellular uptake of the [^99m^Tc]Tc-HYNIC-X conjugates (X: spacer), albeit lower than the corresponding [^99m^Tc]Tc-N4-conjugates. SPECT/CT images showed that all ^99m^Tc-labeled conjugates accumulated in the tumor and kidneys with [^99m^Tc]Tc-HYNIC-PEG_4_-SS01, [^99m^Tc]Tc-N4-SS01 and [^99m^Tc]Tc-N4-JR11 having notably higher kidney uptake. Biodistribution studies showed similar or better tumor-to-non-tumor ratios for the [^99m^Tc]Tc-HYNIC-Ahx conjugates, compared to the [^99m^Tc]Tc-N4 counterparts. The [^99m^Tc]Tc-HYNIC-Ahx conjugates of SS01 and JR11 were comparable to [^99m^Tc]Tc-HYNIC-TOC as imaging agents. HYNIC is a suitable chelator for the development of ^99m^Tc-labeled SST2 antagonists when a spacer of appropriate length, such as Ahx, is used.

## 1. Introduction

Radiolabeled peptide analogs of somatostatin are used for diagnosis and for treatment of neuroendocrine tumors (NETs), given the high expression of somatostatin receptors (SST), especially of subtype 2 (SST2), on the surface of NET cells. The impact of PET imaging with ^68^Ga-labeled SST agonists, such as DOTA-[Tyr^3^]-octreotide and DOTA-[Tyr^3^,Thr^8^]-octreotide (DOTA-TOC and DOTA-TATE, respectively, where DOTA is the chelator 1,4,7,10-tetraazacyclododecane-1,4,7,10-tetraacetic acid), on the management of NET patients has been verified [1]. Preclinical [2,3] and clinical data [4,5] showed that SST antagonists can provide improved diagnostic efficacy over agonists (e.g., OctreoScan^®^, [^68^Ga]Ga-DOTA-TOC or [^68^Ga]Ga-DOTA-TATE).

Despite the increasing application of PET-based radiopharmaceuticals, more than 70% of nuclear medicine procedures still use ^99m^Tc. The ease and cost-effective availability of ^99m^Tc, in combination with the new-generation SPECT scanners with improved spatial resolution and sensitivity, are expected to revive the interest in ^99m^Tc-radiopharmaceuticals. ^99m^Tc-radiopharmaceuticals for imaging NETs are practically based on [Tyr^3^]-octreotide (TOC) conjugated to the monodentate ligand hydrazinonicotinamide (HYNIC) and labeled with ^99m^Tc(V) using ethylenediamine *N*,*N*′ diacetic acid (EDDA) as a co-ligand [6,7,8]. [^99m^Tc]Tc-HYNIC/EDDA-TOC is approved in some European countries (Tektrotyd, NCBJ RC POLATOM). [Tyr^3^,Thr^8^]-octreotide (TATE) was also labeled following the same strategy, and in addition, it was conjugated to the 6-carboxy-1,4,8,11-tetraazaundecane (N4) chelator ([^99m^Tc]Tc-Demotate 1) and used in small cohorts of patients [9,10].

The indications that SST antagonists may have higher sensitivity in imaging NETs than agonists, and the increased interest in ^99m^Tc-radiopharmaceuticals, encouraged us and other groups to develop ^99m^Tc-based SST antagonists for SPECT imaging [11,12,13,14,15,16]. The HYNIC/EDDA and N4 ligands were used, comparatively, for ^99m^Tc-labeling of the SST2 antagonist SS01 (Cpa-c(dCys-Tyr-dTrp-Lys-Thr-Cys)dTyr-NH_2_ [15]. Interestingly, while [^99m^Tc]Tc-N4-SS01 demonstrated high affinity for SST2, [^99m^Tc]Tc-HYNIC/EDDA-SS01 lost affinity completely [15]. This indicated that HYNIC, one of the most commonly used ligands for ^99m^Tc, is unsuitable in combination with SST antagonists, although it demonstrates excellent performance with SST agonists. It remains unclear, however, if this is the case only for SS01 or also for other SST antagonists. In addition, considering that the [^99m^Tc]Tc-N4 conjugates showed very high accumulation in the kidneys [15,16], probably due to the positively charged [^99m^Tc]Tc(O)_2_(N_4_)]^+1^, it is worth investigating if HYNIC can still be used in combination with SST2 antagonists.

In this work, we aimed to investigate whether: 1) the affinity of the HYNIC conjugate [^99m^Tc]Tc-HYNIC/EDDA-SS01 can be restored by incorporating spacers of different lengths and hydrophilicity, as we were aware that SST2 antagonists are extremely sensitive on the N-terminus modifications, often with unexpected outcomes [2]. We chose as spacers the neutral C_3_ β-Alanine (β-Ala), the C_6_ amino-hexanoic acid (Ahx), the C_10_ amino-undecanoic acid (Aun) and the C_11_-type polar and hydrophilic 15-amino-4,7,10,13-tetraoxapentadecanoic acid (PEG_4_). 2) The unsuitability of HYNIC is specific to the analog SS01 or if it is a general phenomenon for SST2 antagonists. For this purpose, we included, in the study, a second SST2 antagonist, namely, JR11 (Cpa-c(dCys-Aph(Hor)-dAph(Cbm)-Lys-Thr-Cys)-dTyr-NH_2_, where Aph(Hor) is 4-amino-phenylalanine(L-hydroorotic acid) and dAph(Cbm) is d-4-amino-phenylalanine(carbamoyl)), known to have high affinity for SST2 [2]. 3) The best [^99m^Tc]Tc-HYNIC-X conjugates (X: spacer) underperform or overperform the corresponding N4 conjugates, namely, [^99m^Tc]Tc-N4-SS01 ([^99m^Tc]Tc-TECANT-2) [15,16] and [^99m^Tc]Tc-N4-JR11. All radiotracers were studied head-to-head in vitro using human embryonic kidney (HEK) cells stably transfected with the human SST2 (HEK-SST2) and in vivo by SPECT/CT imaging in HEK-SST2-positive xenografts. They were all compared with the clinically used [^99m^Tc]Tc-HYNIC/EDDA-TOC (reference radiotracer). Biodistribution and pharmacokinetic studies were performed for the two best performing radiotracers from the series of SS01 and JR11 analogs for the quantification of tumor-to-non-tumor ratios over time. 

## 2. Results

### 2.1. Synthesis, Radiolabeling and Partition Coefficients (log D)

**Figure 1:**
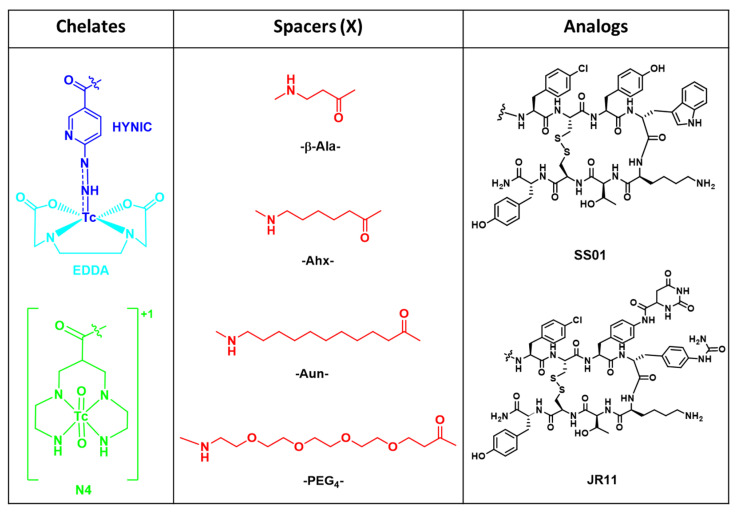
Chemical structure of the SST2 analogs SS01 and JR11, the ^99m^Tc-chelates and the spacers (X).

The chemical structures of the radiolabeled peptide conjugates investigated in this study are shown in Figure 1, and their analytical data are summarized in Table 1. All conjugates were synthesized with a maximum yield of 30–40% by following an Fmoc-based solid phase peptide synthesis. SS01 was coupled either directly to HYNIC or via different spacers, namely, β-Ala, Ahx, Aun and PEG_4_ (Figure 1). JR11 was coupled to HYNIC and HYNIC-Ahx (Figure 1). In addition, both peptides were coupled to the chelator N4 to form the conjugates N4-SS01 and N4-JR11 (Figure 1). The purity of all conjugates was confirmed by RP-HPLC analysis (∼97%) and characterized by ESI-MS (Table 1). All conjugates were labeled with ^99m^Tc with labeling yields of >97% at a maximum apparent molar activity of 130 MBq/nmol. The N4 conjugates were labeled with ^99m^Tc at room temperature within 30 min in the presence of Tin(II)chloride and citrate as an intermediate supporting Tc(V) oxidation state. The HYNIC conjugates were labeled with ^99m^Tc at 95 °C within 15 min in the presence of Tin(II)chloride and EDDA as a co-ligand. For the sake of simplicity, EDDA is omitted from the abbreviated names of the HYNIC-based radiotracers.

The quality control of the radiotracers was repeated after 2 h in their labeling solutions, at pH 7, as described in the materials and methods section. The labeling solutions were kept at room temperature and at activity concentrations in the range of 500–1000 MB/mL for this period. The quality control was performed by radio-HPLC to assess the time period after radiolabeling that the radiotracer can be used safely for evaluation. Most of the radiotracers were found to be stable at room temperature over 2 h, with 95% of intact radiotracer, with the exception of [^99m^Tc]Tc-N4-SS01, [^99m^Tc]Tc-N4-JR11 and [^99m^Tc]Tc-HYNIC-JR11, which were 88 ± 2%, 92 ± 3% and 92 ± 1%, respectively (*n* = 2–3). The radiochemical species found after 2 h were peptide related (radiolytic products). No released ^99m^Tc was detected, while no radical scavengers were added or tested. Based on these results, special care was taken on the use of the three above-mentioned radiotracers immediately after radiolabeling.

The log *D* of the radiotracers was determined by their distribution in phosphate-buffered saline (pH 7.4) and 1-octanol, as described in the experimental section. The results are summarized in Table 1. [^99m^Tc]Tc-HYNIC-Ahx-JR11 exhibited the highest hydrophilicity among all radiotracers (log *D* = −3.15 ± 0.06). In general, all JR11-based radiotracers were more hydrophilic than the corresponding SS01-based radiotracers. The high hydrophobicity of [^99m^Tc]Tc-HYNIC-Aun-SS01 and its adsorption to materials (e.g., tubes, tips and plates) did not enable the reliable determination of its partition coefficient or further evaluation in vitro. [^99m^Tc]Tc-HYNIC-Aun-SS01 was excluded from further studies.

### 2.2. Cellular Distribution

**Figure 2:**
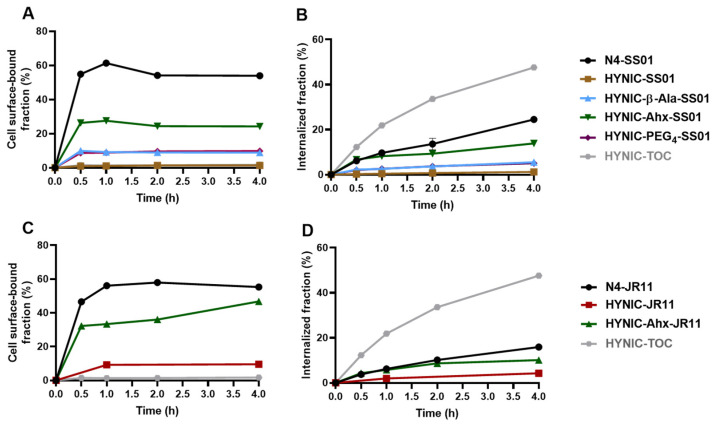
(**A**,**B**) Surface-bound and internalization fractions of all ^99m^Tc-labeled SS01 conjugates. (**C**,**D**) Surface-bound and internalization rates of all ^99m^Tc-labeled JR11 conjugates. For the sake of simplicity, [^99m^Tc]Tc has been omitted from the conjugates. All conjugates were compared to [^99m^Tc]Tc-HYNIC-TOC in HEK-SST2 at 37 °C. Results are expressed as % (mean ± SD) of applied activity in the cells and normalized per million cells. All values refer to specific bound and internalization after subtracting the non-specific (measured in the presence of 1000-fold excess of blocking agent) from the total bound and internalized fractions.

The cell surface-bound and internalized fractions of the radiotracers were assessed in HEK-SST2 cells. Figure 2 depicts the cellular distribution of the SS01-based radiotracers (A and B) and JR11-based radiotracers (C and D), including [^99m^Tc]Tc-HYNIC-TOC, for comparison.

In the series of SS01 conjugates (Figure 2A,B), it was confirmed that while conjugation of N4 leads to high cellular uptake, the conjugation of HYNIC leads to negligible uptake. More precisely, 54.0 ± 0.3% of the applied [^99m^Tc]Tc-N4-SS01 was bound on the cell surface and 24.5 ± 0.3% was internalized after 4 h at 37 °C. Both fractions were almost negligible for [^99m^Tc]Tc-HYNIC-SS01 (1.4 ± 0.1% and 1.2 ± 0.3%, respectively). The introduction of a spacer improved the uptake in all HYNIC conjugates. β-Ala and PEG_4_ had a very similar impact on SS01; the cell surface-bound fraction was 8.9 ± 0.1% and 9.8 ± 1.4% for [^99m^Tc]Tc-HYNIC-β-Ala-SS01 and [^99m^Tc]Tc-HYNIC-PEG_4_-SS01, respectively, and internalized fraction was 5.5 ± 1.0% and 5.1 ± 1.1%, respectively. The spacer Ahx led to the best improvement. [^99m^Tc]Tc-HYNIC-Ahx-SS01 showed a cell surface-bound fraction of 24.2 ± 0.1% and an internalized fraction of 13.9 ± 0.1% at 4 h.

In the series of JR11 conjugates (Figure 2C,D), the HYNIC conjugates followed the same trend, with [^99m^Tc]Tc-HYNIC-Ahx-JR11 showing a much higher cell surface-bound fraction (46.7 ± 0.8% vs. 9.6 ± 0.3%) and internalized fraction (10.1 ± 0.2% vs. 4.28 ± 1.2%) compared to [^99m^Tc]Tc-HYNIC-JR11 at 4 h. Once more, [^99m^Tc]Tc-N4-JR11 performed better; however, both surface-bound (55.3 ± 1.0%) and internalized fractions (15.9 ± 0.5%) were much closer to the values of [^99m^Tc]Tc-HYNIC-Ahx-JR11.

**Figure 3:**
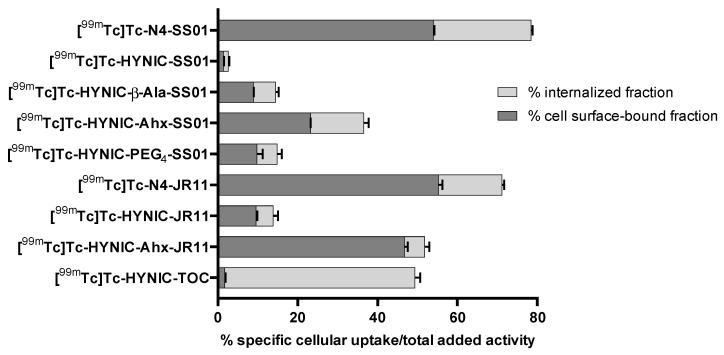
Cellular uptake (internalized fraction in light grey and cell surface-bound fraction in dark grey) of all the radiotracers in HEK-SST2 at 4 h/37 °C. The results are expressed as % (mean ± SD) of applied activity in the cells and normalized per million cells. All values refer to specific internalization and bound after subtracting the non-specific (measured in the presence of 1000-fold excess of blocking agent) from the total internalized and bound fractions.

Figure 3 depicts the results of this study comparing the cellular uptake (cell surface-bound and internalized fractions) of the radiotracers at 4 h at 37 °C.

The highest cellular uptake was exhibited by the two N4 conjugates, [^99m^Tc]Tc-N4-SS01 and [^99m^Tc]Tc-N4-JR11: 78.5 ± 0.3% and 71.2 ± 1.4%, respectively. In the series of HYNIC conjugates, both HYNIC-Ahx conjugates showed superior uptake, compared with all others, with [^99m^Tc]Tc-HYNIC-Ahx-JR11 showing the highest cellular uptake (56.9 ± 0.8%), 1.5 times higher than the corresponding [^99m^Tc]Tc-HYNIC-Ahx-SS01 (38.1 ± 0.2%). Importantly, the cellular uptake of the reference radiotracer [^99m^Tc]Tc-HYNIC-TOC was measured at a very similar level (49.4 ± 1.2%), with the main difference being the cellular distribution; as an agonist, it was internalized almost entirely (47.6 ± 1.3%; cell surface-bound fraction: 1.7 ± 0.2%). On the basis of the cellular uptake, the radiotracers [^99m^Tc]Tc-N4-SS01, [^99m^Tc]Tc-HYNIC-Ahx-SS01, [^99m^Tc]Tc-N4-JR11 and [^99m^Tc]Tc-HYNIC-Ahx-JR11 were selected for further in vitro assessment.

### 2.3. Kinetics of Association on HEK-SST2 Cell Membranes and Related Saturation Binding

**Figure 4:**
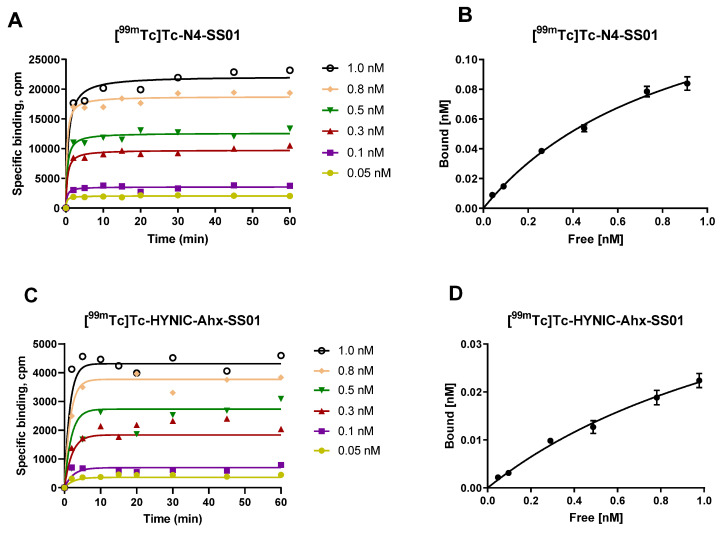

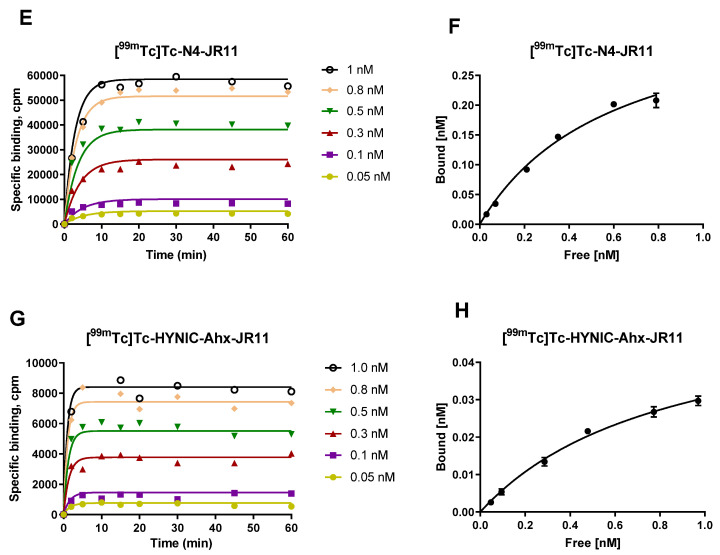
(**A**,**C**,**E**,**G**) Association profiles of the four radiotracers obtained by incubating the HEK-SST2 cell membrane (10 µg/well) with the indicated concentrations of corresponding radiotracer over 60 min. Data were fitted using the one-phase exponential association equation. (**B**,**D**,**F**,**H**) Saturation binding profiles for the radioligands obtained by association data**.** Data were treated with the equation: one site, specific binding (GraphPad, Software Inc., Prism 7).

The kinetics of interaction among [^99m^Tc]Tc-N4-SS01, [^99m^Tc]Tc-HYNIC-Ahx-SS01, [^99m^Tc]Tc-N4-JR11 and [^99m^Tc]Tc-HYNIC-Ahx-JR11 with the receptor on the cell membrane were fast and similar (Figure 4A,C,E,G). Equilibrium was reached between 10 and 20 min. The bound fractions, between 20 and 60 min for each individual concentration, were treated to plot the corresponding saturation curves (Figure 4B,D,F,H).

The affinities of all four radiotracers were in the low nanomolar range and very similar for [^99m^Tc]Tc-N4-SS01, [^99m^Tc]Tc-N4-JR11 and [^99m^Tc]Tc-HYNIC-Ahx-JR11 (K_D_ = 0.92 ± 0.16, 0.60 ± 0.14 and 0.85 ± 0.14 nM, respectively), with the exception of [^99m^Tc]Tc-HYNIC-Ahx-SS01, which had an approximately two-fold lower affinity (K_D_ = 1.60 ± 0.46 nM), but this is still very high for SST2.

A higher number of binding sites was recognized by [^99m^Tc]Tc-N4-JR11, followed by [^99m^Tc]Tc-N4-SS01 (B_max_ = 0.38 ± 0.05 and 0.17 ± 0.02 nM, respectively), while the two [^99m^Tc]Tc-HYNIC-Ahx conjugates recognized the same number of binding sites (B_max_ = 0.06 ± 0.01 and 0.06 ± 0.005 nM for [^99m^Tc]Tc-HYNIC-Ahx-SS01 and [^99m^Tc]Tc-HYNIC-Ahx-JR11, respectively).

### 2.4. SPECT/CT Imaging

**Figure 5:**
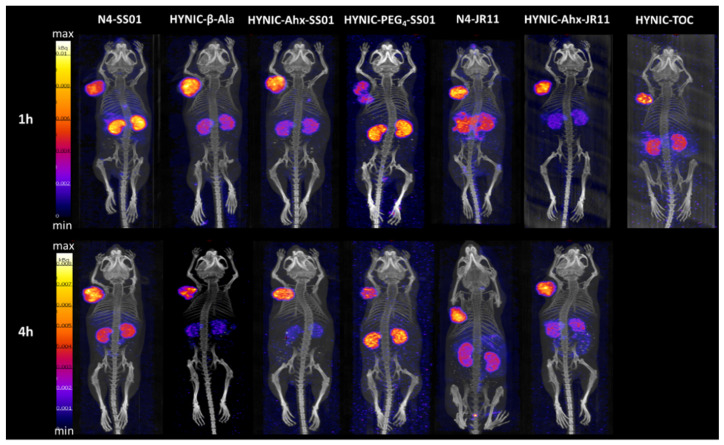
SPECT/CT images of all [^99m^Tc]Tc-labeled conjugates at 1 and 4 h p.i. The mice bearing HEK-SST2 tumors were intravenously injected with 100 µL/200 pmol/ 4–6 MBq of the indicated ^99m^Tc-labeled conjugate and were euthanized 1 or 4 h p.i. by keeping them in a CO_2_ chamber for 2 min, followed by a slow increase in the concentration of CO_2_ gas.

The SPECT/CT images were acquired head-to-head for all radiotracers at 1 and 4 h post injection (p.i.) (Figure 5). Imaging studies were not performed for [^99m^Tc]Tc-HYNIC-SS01 or [^99m^Tc]Tc-HYNIC-JR11, as the in vitro data revealed very low cellular uptake, nor for [^99m^Tc]Tc-HYNIC-Aun-SS01, which was excluded for the reasons described above.

All the tested radiotracers were able to visualize SST2-expressing tumors and were mainly accumulated in the kidneys, with the exception of [^99m^Tc]Tc-N4-JR11 at 1 h p.i., additionally showing some abdominal uptake (attributed to stomach and pancreas). [^99m^Tc]Tc-HYNIC-PEG_4_-SS01 showed the highest kidney uptake and lowest tumor uptake at 4 h p.i. among all radiotracers. While [^99m^Tc]Tc-HYNIC-β-Ala-SS01 demonstrated very low kidney uptake at 4 h p.i., it also showed very fast washout from the tumor. The two [^99m^Tc]Tc-N4 conjugates demonstrated higher kidney uptake than the other radiotracers, with the exception of [^99m^Tc]Tc-HYNIC-PEG_4_-SS01. The two [^99m^Tc]Tc-HYNIC-Ahx conjugates exhibited visually similar tumor uptake and lower kidney uptake compared to their [^99m^Tc]Tc-N4 counterparts and similar or superior image contrast as compared to [^99m^Tc]Tc-HYNIC-TOC.

On the basis of the SPECT/CT images, and of the in vitro cellular uptake, the radiotracers [^99m^Tc]Tc-HYNIC-Ahx-SS01 and [^99m^Tc]Tc-HYNIC-Ahx-JR11 were selected for further in vivo evaluation, in comparison to their [^99m^Tc]Tc-N4 counterparts.

### 2.5. Biodistribution Studies

Biodistribution studies of [^99m^Tc]Tc-N4-SS01, [^99m^Tc]Tc-HYNIC-Ahx-SS01, [^99m^Tc]Tc-N4-JR11 and [^99m^Tc]Tc-HYNIC-Ahx-JR11 were performed on HEK-SST2 tumor-bearing mice at 1, 4 and 24 h p.i. (Table 2 and Table 3).

In the case of the SS01 analogs (Table 2), tumor uptake was similar for both [^99m^Tc]Tc-HYNIC-Ahx-SS01 and [^99m^Tc]Tc-N4-SS01 (14.94 ± 5.15 and 19.12 ± 4.47 %IA/g, respectively; *p* = 0.266) at 1 h p.i. However, at 4 h p.i., [^99m^Tc]Tc-HYNIC-Ahx-SS01 demonstrated significantly lower tumor uptake (12.82 ± 3.09 and 28.41 ± 4.84 %IA/g; *p* = 0.002) and remained low at 24 h p.i. (3.82 ± 0.83 and 10.98 ± 4.69 %IA/g; *p* = 0.06). On the other hand, uptake in the SST2-expressing organs—non-tumor tissues, kidneys and bloodstream—was significantly lower for [^99m^Tc]Tc-HYNIC-Ahx-SS01 than for [^99m^Tc]Tc-N4-SS01. Consequently, the tumor-to-background ratios were favorable for [^99m^Tc]Tc-HYNIC-Ahx-SS01 at all investigated time points, despite the higher tumor uptake of [^99m^Tc]Tc-N4-SS01.

A similar trend was found for the JR11 analogs (Table 3). [^99m^Tc]Tc-HYNIC-Ahx-JR11 and [^99m^Tc]Tc-N4-JR11 showed similar tumor uptake at 1 h p.i. (14.22 ± 1.25 and 16.41 ± 2.87 %IA/g, respectively; *p* = 0.213), while [^99m^Tc]Tc-HYNIC-Ahx-JR11 showed lower uptake at 4 (13.49 ± 2.07 and 19.07 ± 1.83 %IA/g; *p* = 0.007) and 24 h p.i. (5.83 ± 0.43 and 12.29 ± 1.59 %IA/g; *p* = 0.002). The difference between the two JR11-based radiotracers in total body distribution also followed the same trend, with lower background activity for [^99m^Tc]Tc-HYNIC-Ahx-JR11 but not as profound as in the case of the SS01-based radiotracers. Consequently, superiority was not observed in one vs. another radiotracer in terms of tumor-to-non-tumor ratios.

## 3. Discussion

A number of preclinical and initial clinical studies (summarized in [17,18]) suggested improvements in both diagnostic sensitivity and therapeutic efficacy by the use of radiolabeled SST2 antagonists (i.e., DOTA-JR11) instead of agonists (i.e., DOTA-TATE or DOTA-TOC). This was attributed to different reasons, including but not limited to, the following: (a) a higher number of binding sites recognized by the antagonists on the surface of SST-expressing cells [19,20], with consequently higher accumulation; (b) lower uptake of the antagonists in organs that were the primary sites of metastasis (i.e., liver), enabling better lesion-to-background contrast, and as such better image sensitivity [4]; (c) longer retention of the antagonists in the tumor, leading to higher tumor radiation doses [3,21]; and (d) a higher number of DNA double strand breaks caused by the antagonists on SST-expressing cells [22], suggesting more effective treatment. All these findings indicate that internalization, which is induced upon the binding of an agonist to the receptor but not by an antagonist, is not a prerequisite for high image contrast or for effective treatment, as it was initially assumed.

The choice of the somatostatin analog, i.e., agonist or antagonist; the presence of spacers; the chelating system; and the radiometal play an important role in modulating receptor binding affinity and in vivo distribution [2,23,24,25]. A remarkable example was reported by Abiraj et al. [15] on the somatostatin antagonist SS01, showing drastically different binding properties when conjugated to two chelating systems widely used for radiolabeling with ^99m^Tc. The complete loss of affinity of [^99m^Tc]Tc-HYNIC-SS01 for SST2, which could be restored using the acyclic tetramine N4 ([^99m^Tc]Tc-N4-SS01), warranted further investigation. This work intended to investigate the role of the HYNIC as a monodentate ligand for ^99m^Tc-labeled SST2 antagonists and the influence of different spacers on the modulation of the binding affinity of the HYNIC conjugates. In addition, we aimed to determine the suitability of known chelating systems for the development of ^99m^Tc-labeled SST2 antagonists.

The introduction of spacers between the chelator and the peptide moiety is a common strategy to prevent steric influence and to retain the affinity of the peptide for its receptor [26]. This was not required when SS01 was conjugated to N4 ([^99m^Tc]Tc-N4-SS01) or to DOTA ([^177^Lu]Lu-DOTA-SS01) [15]. However, it was shown that SST2 antagonists are very sensitive to N-terminus modifications, with different chelating systems and radiometals having unexpected impacts on affinity, biodistribution and pharmacokinetics [2,27]. This seemed to be the case for the HYNIC vs. N4 conjugates of the SST2 antagonists. A significant difference between the two is the nature of the ^99m^Tc-complex. N4 is labeled via the Tc-dioxo core forming the positively charged [^99m^Tc(O)_2_(N_4_)]^+1^ moiety (Figure 1). HYNIC is labeled via the Tc(V)-hydrazino metal fragment ([^99m^Tc=N=N–R]^2+^), where it can be coordinated as a diazene (neutral) or diazenido (anionic) ligand, with EDDA acting as a co-ligand to stabilize the coordination geometry (Figure 1). The structure of the Tc primary coordination sphere in the case of HYNIC is uncertain. [^99m^Tc]Tc-HYNIC complexes have different isomeric forms, and the corresponding radiotracers adapt various conformations, without well-defined chemical structures [28,29,30]. We hypothesized that certain conformations of the HYNIC conjugates [^99m^Tc]Tc-HYNIC-SS01 and [^99m^Tc]Tc-HYNIC-JR11 were responsible for the loss of affinity and that they may be altered when introducing spacers. Unfortunately, the unclear chemistry around [^99m^Tc]Tc-HYNIC complexes and the difficulty to obtain a non-radioactive chemically identical compound (e.g., ^99g^Tc-HYNIC/EDDA-SS01) limited the options of investigating this hypothesis by docking or NMR studies.

We confirmed the low cellular uptake of [^99m^Tc]Tc-HYNIC-SS01, reported by Abiraj et al. [15], and were able to identify that introducing a spacer restores, to different extents depending on the spacer’s length and physicochemical properties, the binding affinity of SS01 toward SST2. The neutral C_3_ β-Ala and C_6_ Ahx and the polar and hydrophilic C_11_-type PEG_4_ led to a significant improvement in the SST2-mediated cellular uptake in vitro, with the C_6_ distance allowing the best binding to the receptor. The aliphatic C_10_ Aun was proven to be an unsuitable spacer due to its hydrophobicity and adsorption to materials. Therefore, [^99m^Tc]Tc-HYNIC-Aun-SS01 was excluded from the study. More specifically, the introduction of a C_3_ spacer ([^99m^Tc]Tc-HYNIC-β-Ala-SS01) improved the cellular uptake of [^99m^Tc]Tc-HYNIC-SS01 by a factor of six (approx. 15 vs. 2.5%, respectively, at 4 h). Increasing the length to C_6_ ([^99m^Tc]Tc-HYNIC-Ahx-SS01) increased the cellular uptake nearly to the levels of [^99m^Tc]Tc-HYNIC-TOC (approx. 40 vs. 50%, respectively, at 4 h). Further increasing the length, while introducing polar hydrophilic properties ([^99m^Tc]Tc-HYNIC-PEG_4_-SS01), did not lead to any further improvement. On the contrary, cellular uptake was at the level of the β-Ala conjugate (approx. 15%). In summary, [^99m^Tc]Tc-HYNIC-Ahx-SS01 increased the cellular uptake of [^99m^Tc]Tc-HYNIC-SS01 by 16-fold, though it remained significantly lower than the uptake achieved with [^99m^Tc]Tc-N4-SS01 (38.1 ± 0.2 vs. 78.5 ± 0.3%, respectively, at 4 h).

The influence of the spacers was also illustrated in the SPECT/CT images: the β-Ala conjugate showed high tumor and low kidney uptake at 1 h p.i., yet very fast washout from the tumor from 1 to 4 h p.i. The PEG_4_ conjugate was characterized by high kidney uptake and rather low accumulation in the tumor, compared to all other derivatives, despite the similar cellular uptake to the β-Ala conjugate in vitro. We observed that the in vivo tumor uptake was better correlated to the affinity (K_D_) and B_max_ in vitro data (measured at concentrations at saturation level) than to the cellular uptake determined at sub-saturation levels. The Ahx conjugate showed high and persistent accumulation in the tumor, lower, however, than [^99m^Tc]Tc-N4- SS01 (14.94 ± 5.15 and 12.82 ± 3.09 %IA/g at 1 and 4 h p.i., respectively, for the HYNIC-Ahx conjugate vs. 19.12 ± 4.47 and 28.41 ± 4.84 %IA/g at 1 and 4 h p.i., respectively, for the N4 conjugate), as confirmed by quantitative biodistribution studies. The difference between these two conjugates may be attributed to the lower number of binding sites recognized by the HYNIC-Ahx vs. the N4 conjugate (B_max_ = 0.06 ± 0.01 vs. 0.17 ± 0.02 nM, respectively) and/or to the slightly lower affinity (K_D_ = 1.60 ± 0.46 vs. 0.92 ± 0.16 nM, respectively). On the other hand, the HYNIC-Ahx conjugate showed significantly lower kidney retention (9.47 ± 1.74 and 5.60 ± 0.47 %IA/g at 1 and 4 h p.i.) compared to the N4 conjugate (43.63 ± 11.37 and 25.85 ± 5.23 %IA/g at 1 and 4 h p.i.). The kidney uptake was washed out faster than the tumor, leading to a tumor-to-kidney ratio of 2.3 at 4 h p.i. for [^99m^Tc]Tc-HYNIC-Ahx-SS01, which was higher than that achieved with [^99m^Tc]Tc-N4-SS01 (T/K = 1.1 at 4 h p.i., recently published as [^99m^Tc]Tc-TECANT-2 [16]). Interestingly, the lower kidney and background activity of [^99m^Tc]Tc-HYNIC-Ahx-SS01, compared to [^99m^Tc]Tc-N4-SS01, favored [^99m^Tc]Tc-HYNIC-Ahx-SS01 in terms of tumor-to-background contrast, despite the significantly higher tumor uptake of [^99m^Tc]Tc-N4-SS01. The image contrast of [^99m^Tc]Tc-HYNIC-Ahx-SS01 compared well with [^99m^Tc]Tc-HYNIC-TOC in SPECT/CT imaging at 1 h p.i.

The choice of JR11 as an alternative SST2 antagonist has been dictated by the presence of innumerable data on this peptide, conjugated with different chelators and radiolabeled with various radiometals. It is currently being evaluated in clinical trials (known as OPS201 or [^177^Lu]Lu-DOTA-JR11 and as OPS202 or [^68^Ga]Ga-NODAGA-JR11) (summarized in [18]). The trend observed in the SS01 family was the same for the conjugates of the JR11 family, although [^99m^Tc]Tc-HYNIC-JR11 showed non-negligible cellular uptake (approx. 15% at 4 h), which was not the case for [^99m^Tc]Tc-HYNIC-SS01. The introduction of Ahx increased the cellular uptake by a factor of four (approx. 55%), while once more, the highest cellular uptake was found for [^99m^Tc]Tc-N4-JR11 (approx. 70%).

The SPECT/CT images of [^99m^Tc]Tc-HYNIC-Ahx-JR11 vs. [^99m^Tc]Tc-N4-JR11 showed differences in kidney uptake, as seen for SS01, as well as in abdominal uptake, with [^99m^Tc]Tc-N4-JR11 having higher accumulation in both. The quantitative biodistribution data showed that both radiotracers had similar tumor uptake at 1 h p.i. (14.22 ± 1.25 and 16.41 ± 2.87 %IA/g for [^99m^Tc]Tc-HYNIC-Ahx-JR11 and [^99m^Tc]Tc-N4-JR11, respectively). This was in agreement with their similar affinities (K_D_ = 0.85 ± 0.14 nM and 0.60 ± 0.14 nM for the HYNIC-Ahx and N4 conjugates, respectively) and with their cellular uptake (approx. 60 and 70%, respectively), although the B_max_ of [^99m^Tc]Tc-HYNIC-Ahx-JR11 (0.06 ± 0.005 nM) was lower than that of [^99m^Tc]Tc-N4-JR11 (0.38 ± 0.05 nM). [^99m^Tc]Tc-HYNIC-Ahx-JR11 washed out faster from the tumor, but its lower background activity balanced the faster tumor washout, leading to very similar tumor-to-background contrast for both radiotracers in most of the cases. The abdominal uptake observed for [^99m^Tc]Tc-N4-JR11 was also seen in the biodistribution profile of DOTA-JR11 labeled with either ^177^Lu, ^111^In or ^90^Y [3]. The biodistribution profile of [^99m^Tc]Tc-N4-JR11 was comparable to the recently published [^99m^Tc]Tc-TECANT-1 (= [^99m^Tc]Tc-N4-LM3, where LM3: p-Cl-Phe-cyclo(d-Cys-Tyr-d-4-amino-Phe(carbamoyl)-Lys-Thr-Cys)-d-Tyr-NH_2_ [27]). [^99m^Tc]Tc-TECANT-1 is the first ^99m^Tc-based SST2 antagonists selected for clinical translation, and it is entering clinical evaluation within the ERA-PerMED project “TECANT” [16].

In summary, although we could not elucidate the mechanism behind the influence of the spacer in HYNIC conjugates of SST2 antagonists, we could show that the introduction of spacers has a significant impact on this class of radiotracers. This is clinically relevant, first because there are indications that the ^99m^Tc-based SST2 antagonist may improve NET diagnostics with SPECT, similarly to ^68^Ga-based SST2 antagonists for PET [4], and second because there is already an approved ^99m^Tc-radiopharmaceutical based on the HYNIC strategy ([^99m^Tc]Tc-HYNIC-TOC, Tektrotyd), while there is no approved radiopharmaceutical based on N4.

## 4. Materials and Methods

The Nα-Fmoc-protected amino acids and Rink Amide resin (0.36 g/mol) were procured from Merck Novabiochem (Darmstadt, Germany). DOTA(^t^Bu)_3_ was obtained from CheMatech (Dijon, France). Succinimidyl-N-Boc-HYNIC was purchased from ABX Advanced Biochemical Compounds (Radeberg, Germany). All other reagents and solvents were purchased from Acros Organics (Geel, Belgium) and Sigma Aldrich (Buchs, Switzerland) and used without further purification. For radiolabeling, [^99m^Tc]NaTcO_4_ was eluted in normal saline from a commercial ^99^Mo/^99m^Tc generator (Mallinckrodt Medical B.V., Petten, The Netherlands). Quantitative γ-counting was carried out on a COBRA 5003 γ-system well counter from Packard Instruments (Meriden, CT, USA). Liquid chromatography mass spectrometry (LC–MS) was run on a LCMS-2020 SHIMADZU system equipped with a Waters XBridge C-18 column (4.6 × 150 mm, 5 µm particle size). Eluents: A = H_2_O (0.1% TFA); B = acetonitrile (0.1% TFA); gradient: 15-65% solvent B in 15 min; flow rate: 1.0 mL/min.

### 4.1. Synthesis of the Chelator-Peptide Conjugates

The peptide SS01 was assembled on the automated microwave peptide synthesizer Liberty Blue (CEM, Charlotte, NC, USA) using Rink-Amide methylbenzylhydryl (MBHA) resin. The spacers, Fmoc-Ahx-OH, Fmoc-Aun-OH and Fmoc-PEG_4_-OH, and the succinimidyl-N-Boc-HYNIC were then coupled manually to afford the desired peptide conjugate derivatives. The conjugate HYNIC-Ahx-JR11 was synthesized following the same protocol as above. The peptides were finally purified on a Bischoff chromatography system by semi-preparative RP-HPLC using a waters XBridge C-18 column (10 mm × 150 mm, 5 µm particle size). Eluents: A = H_2_O (0.1% TFA) and B = acetonitrile (0.1% TFA); gradient: 5 to 50% B in 15 min; flow rate: 2 mL/min. The conjugates HYNIC-β-Ala-SS01, N4-SS01, HYNIC-JR11 and N4-JR11 were purchased from PiChem (Austria). HYNIC-TOC was kindly provided by POLATOM (Poland). Purity and identity were confirmed by LC–MS. The analytical data of the peptide conjugates are reported in Table 1.

### 4.2. Synthesis of the Radiolabeled Conjugated and Stability

^99m^Tc-HYNIC conjugates: A total of 5-11 nmol of the corresponding HYNIC peptide was added to a mixture of 250 µL of Tricine (80 mg/mL) and 250 µL of EDDA (20 mg/mL) dissolved in 0.1 M NaOH. To this mixture, 150–400 MBq of [^99m^Tc]NaTcO_4_ and 5 µL of SnCl_2_ in 0.1 M HCl (2 mg/mL) were added and incubated for 15 min at 95 °C. The pH of the solution was checked (pH 7). Quality control was performed by radio-HPLC.

^99m^Tc-N4 conjugates: An amount of 6-10 nmol of the corresponding N4 peptide was mixed in a solution containing 25 µL of 0.5 M phosphate buffer (pH 11.5) and 5 µL of 0.1 M sodium citrate. To this mixture, 100-400 MBq of [^99m^Tc]NaTcO_4_ and 5 µL of freshly prepared SnCl_2_ (2 mg/mL) solution in ethanol were added. The reaction mixture was incubated at room temperature for 30 min and later neutralized by adding 5 µL of 1 M NaH_2_PO_4_.

Quality control of all radiotracers was performed by analytical RP-HPLC on a Bischoff LC-CaDi 22-14 interface with a UV-vis Lambda 1010 detector and a flow-through Berthold LB509 γ-detector using a Phenomenex Jupiter Proteo 90 Å C12 (250 × 4.6 mm) column. (Eluents: A = H_2_O (0.1% TFA); B = acetonitrile (0.1% TFA); gradient: 95–50% solvent A in 15 min; flow rate: 2 mL/min.)

The stability of all radiotracers at room temperature was assessed up to 2 h by radio-HPLC.

### 4.3. Log D Measurements

Determination of log *D_(_*_pH = 7.4)_ was performed by the shake-flask method. In a pre-lubricated Eppendorf tube, a pre-saturated mixture of 500 µL 1-octanol and 500 µL of phosphate-buffered saline (PBS) at pH 7.4 was added. An amount of 10 µL of 1 µM radiotracer solution was added to this mixture, followed by vortexing of the tube at room temperature for 15 min to reach equilibrium. Centrifugation for 15 min at 3000 rpm was performed for separation of the phases. Aliquots of 100 µL were removed from the octanol and from the PBS phase, and the activity measured in a γ-counter. The partition coefficient was calculated as the average log ratio value of the radioactivity in the organic fraction and PBS fraction.

### 4.4. In Vitro Characterization

#### 4.4.1. Cell Cultures and Cell Membranes

The HEK-293 cell line expressing the T7-epitope tagged human SST2 receptor (HEK-SST2) was provided by Prof. Stefan Schulz (Institute of Pharmacology and Toxicology, Jena University Hospital, Jena, Germany). HEK-SST2 cells were cultured at 37 °C and 5% CO_2_ in DMEM containing 10% fetal bovine serum (FBS), 100 U/mL penicillin, 100 μg/mL streptomycin, 200 µmol/mL l-Glutamin and 500 μg/mL G418. For all the in vitro assays on intact cells, HEK-SST2 cells were seeded into six-well plates (about 1.0 × 10^6^ cells) 24 h before starting the experiment, incubated with 1% FBS containing medium at 37 °C/5% CO_2_. On the day of the experiment, the cells were washed and incubated with the adjusted medium volume for 1 h prior to starting the experiment. The plates were pre-treated with a solution of 10% poly-lysine to promote cell attachment.

For cell membrane preparation, the HEK-SST2 cells were grown to confluence, mechanically disaggregated, washed with PBS buffer (pH 7.0) and re-suspended in homogenization buffer (Tris 20 mM; ethylenediaminetetraacetic acid (EDTA) 1 mM; sucrose 0.25 M; bacitracin 1 mg/mL; soybean trypsin inhibitor 0.1 mg/mL; and phenylmethylsulfonyl fluoride (PMSF) 0.125 mg/mL, pH 7.5). Cells were homogenized using Ultra-Turrax, and the homogenized suspension was centrifuged at 500 g for 10 min at 4 °C. The supernatant was collected in centrifuge tubes (Beckman Coulter Inc., Brea, CA, USA), and this procedure was repeated five times. The collected supernatant was centrifuged in an ultra-centrifuge (Beckman) at 4 °C for 55 min at 20,000 RPM. Then, the pellet was re-suspended in ice-cold 4-(2-hydroxyethyl)-1-piperazineethanesulfonic acid (HEPES) buffer (10 mM, pH 7.5), aliquoted and stored at −80 °C. The protein concentration of the membrane solution was determined using the Bradford method with bovine serum albumin as the standard.

#### 4.4.2. In Vitro Internalization

The cell surface-bound and internalization rates of the radiotracers were studied in HEK-SST2 cells seeded in six-well plates, as described previously [27]. Briefly, the radiotracer (2.5 nM) was added, and the cells were incubated at 37 °C. At different time points (0.5, 1, 2 and 4 h), cellular uptake was stopped by washing twice with ice-cold PBS. A membrane-bound radiotracer was obtained by washing cells twice with ice-cold glycine buffer (pH 2.8), followed by a collection of the internalized fraction with 1 M NaOH. The activity in each fraction was measured in a γ-counter (Cobra II). Non-specific uptake was determined in the presence of 1000-fold excess of the corresponding conjugate. The results were expressed as a percentage of the applied radioactivity, after subtracting the non-specific uptake.

#### 4.4.3. Kinetics of the Association on HEK-SST2 Cell Membranes and Related Saturation Binding

The association profiles of [^99m^Tc]Tc-N4-SS01, [^99m^Tc]Tc-HYNIC-Ahx-SS01, [^99m^Tc]Tc-N4-JR11 and [^99m^Tc]Tc-HYNIC-Ahx-JR11 were studied at different concentrations, ranging from 0.05 to 1 nM, in HEK-SST2 cell membranes at 37 °C. Each assay tube contained the following: 170 μL binding buffer (20 ×HEPES, pH 7.4, containing 4 mM MgCl_2_, 0.2% BSA, 20 mg/L bacitracin, 20 mg/L PMSF and 200,000 KIU/L aprotinin), 30 μL radiotracer solution of the corresponding concentration and 100 μL cell membrane suspension (10 μg protein content). For the non-specific series, instead of 170 μL binding buffer, 140 μL binding buffer and 30 μL of blocking agents were added. The bound fraction was measured at different time points, starting from 2 up to 60 min. Equilibrium was reached after 20 min of incubation, and the bound fractions between 20 and 60 min were averaged and plotted vs. their corresponding concentrations to graph the saturation binding curves.

### 4.5. In Vivo Evaluation

#### 4.5.1. Tumor Implantation

The Veterinary Office (Department of Health) of the Cantonal Basel-Stadt approved the animal experiments (approval no. 30515) in accordance with the Swiss regulations for animal treatment. Female athymic nude-Foxn1nu/Foxn1+ mice (Envigo, The Netherlands), 4–6 weeks old, were injected subcutaneously with 10^7^ HEK-SST2 cells in the right shoulder, freshly suspended in 100 μL sterile phosphate-buffered saline. The tumors were allowed to grow for 2–3 weeks.

#### 4.5.2. SPECT/CT Imaging

Mice bearing HEK-SST2 tumors were euthanized 1 or 4 h after intravenous injection of 4–6 MBq (200 pmol) of the ^99m^Tc-labeled conjugates and imaged supine, head first, using a SPECT/CT system dedicated to imaging small animals (NanoSPECT/CT^TM^ Bioscan Inc.). Topograms and helical CT scans of the whole mouse were first acquired using the following parameters: X-ray tube current: 177 µA, X-ray tube voltage 45 kVp, 90 s and 180 frames per rotation, pitch 1. The helical SPECT scan was then acquired from head to toe using multi-purpose pinhole collimators (APT1). The energy window width was 20%, centered symmetrically over the energy peak of ^99m^Tc at 140 keV. Twenty-four projections (200 s per projection) were used, allowing acquisition of at least 50 kilocounts/projection.

SPECT images were reconstructed iteratively and filtered using the HiSPECT software package (version 1.4.1876, SciVis GmbH, Goettingen, Germany) and the manufacturer’s algorithm (3 subsets, 9 iterations, 35% postfiltering, 128 × 128 matrix, zoom 1, 30 × 20 mm transaxial field of view, resulting in a pixel size of 0.3 mm). CT images were reconstructed using CTReco (version r1.146), with a standard filtered back projection algorithm (exact cone beam) and postfiltered (RamLak, 100% frequency cut-off), resulting in a pixel size of 0.2 mm. Co-registered images were visualized in the three orthogonal planes using maximum intensity projection with InVivoScope (version 1.43, Bioscan Inc.).

#### 4.5.3. Biodistribution and Pharmacokinetics

The radiotracers [^99m^Tc]Tc-N4-SS01, [^99m^Tc]Tc-HYNIC-Ahx-SS01, [^99m^Tc]Tc-N4-JR11 and [^99m^Tc]Tc-HYNIC-Ahx-JR11 were injected via the tail vein (100 µL, 20 pmol, 0.6–0.8 MBq) and sacrificed at 1, 4 and 24 h p.i. Organs of interest and blood were collected, rinsed of excess blood, blotted dry, weighed and counted in a γ-counter. The percentage of injected activity per gram (%IA/g) was calculated for each tissue. The total counts injected per animal were determined by extrapolation from counts of an aliquot taken from the injected solution as a standard.

## 5. Conclusions

Two highly potent SST2 antagonists, SS01 and JR11, were selected and conjugated to HYNIC, with and without different spacers, and to N4 as chelating systems for ^99m^Tc-labeling and studied in vitro and in vivo. The conjugation of HYNIC significantly hindered the binding properties of both SS01 and JR11, but the introduction of a spacer remedied this obstacle. HYNIC proved to be a suitable chelating system for the development of ^99m^Tc-labeled SST2 antagonists when a spacer of appropriate length, such as Ahx, is used, and the suitability of N4 was confirmed. When comparing HYNIC-Ahx to the N4 conjugates, while the HYNIC-Ahx conjugates showed faster washout from the tumor, they were either better (in the case of SS01) or equivalent (in the case of JR11) to the N4 conjugates, in terms of tumor-to-background contrast. [^99m^Tc]Tc-HYNIC-Ahx-SS01 and [^99m^Tc]Tc-HYNIC-Ahx-JR11 compare well with [^99m^Tc]Tc-HYNIC-TOC as imaging agents.

## Figures and Tables

**Table 1:** Analytical data of studied conjugates and log *D*_(pH = 7.4)_ of corresponding ^99m^Tc-labeled conjugates.

Conjugate	*m*/*z* Calcd.	*m*/*z* Meas.	t_R_ (min) ^a^	Log *D*_(pH = 7.4)_^b^
N4-SS01	1331.01	1331.35	10.5	−2.49 ± 0.34 ^c^
HYNIC-SS01	1279.88	1279.46	12.2	−2.21 ± 0.07
HYNIC-β-Ala-SS01	1350.95	1350.95	11.8	−1.91 ± 0.19
HYNIC-Ahx-SS01	1393.04	1394.76	12.4	−1.96 ± 0.06
HYNIC-Aun-SS01	1463.18	1463.61	14.7	n.d.
HYNIC-PEG_4_-SS01	1525.59	1526.36	12.5	−2.03 ± 0.13
N4-JR11	1489.12	1488.58	9.6	−2.80 ± 0.19
HYNIC-JR11	1437.99	1437.52	11.0	−2.88 ± 0.11
HYNIC-Ahx-JR11	1551.15	1551.5	11.2	−3.15 ± 0.06
HYNIC-TOC	1170.36	1170.53	11.1	−2.77 ± 0.07

^a^ t_R_, retention time; ^b^ log *D*, partition coefficient between 1-octanol and PBS (pH 7.4) for corresponding ^99m^Tc-labeled conjugates; n.d., not determined; ^c^ from [15].

**Table 2:** Biodistribution studies of [^99m^Tc]Tc-N4-SS01 and [^99m^Tc]Tc-HYNIC-Ahx-SS01 in SST2-expressing xenografts at 1 (*n* = 4), 4 (*n* = 4) and 24 (*n* = 3) hours p.i. Results are expressed as mean of the % injected activity per gram of tissue (% IA/g) ± standard deviation (SD).

Organ	[^99m^Tc]Tc-N4-SS01 ^a^			[^99m^Tc]Tc-HYNIC-Ahx-SS01		
	1 h	4 h	24 h	1 h	4 h	24 h
**Blood**	1.80 ± 0.44	0.23 ± 0.05	0.03 ± 0.01	0.85 ± 0.12	0.10 ± 0.02	0.01 ± 0.004
**Heart**	1.64 ± 0.60	0.26 ± 0.05	0.05 ± 0.01	0.45 ± 0.08	0.07 ± 0.00	0.02 ± 0.004
**Lung**	17.00 ± 5.75	2.52 ± 0.53	0.30 ± 0.05	2.45 ± 0.56	0.32 ± 0.05	0.06 ± 0.01
**Liver**	5.38 ± 1.36	3.72 ± 0.92	1.07 ± 0.24	1.48 ± 0.26	0.44 ± 0.08	0.21 ± 0.06
**Pancreas**	16.37 ± 4.53	1.96 ± 0.58	0.12 ± 0.05	4.19 ± 1.08	0.27 ± 0.08	0.03 ± 0.003
**Spleen**	1.61 ± 0.46	0.55 ± 0.15	0.24 ± 0.06	0.51 ± 0.09	0.16 ± 0.03	0.07 ± 0.02
**Stomach**	14.60 ± 4.89	2.17 ± 0.39	0.45 ± 0.08	2.69 ± 0.39	0.59 ± 0.16	0.09 ± 0.02
**Intestine**	4.05 ± 1.12	0.86 ± 0.40	0.31 ± 0.17	0.91 ± 0.33	0.26 ± 0.13	0.03 ± 0.01
**Adrenal**	5.14 ± 1.79	1.25 ± 0.33	0.73 ± 0.20	0.74 ± 0.13	0.30 ± 0.11	0.04 ± 0.03
**Kidney**	43.63 ± 11.37	25.85 ± 5.23	3.42 ± 1.44	9.47 ± 1.74	5.60 ± 0.47	1.15 ± 0.15
**Muscle**	0.69 ± 0.22	0.15 ± 0.04	0.06 ± 0.03	0.24 ± 0.08	0.05 ± 0.01	0.01 ± 0.004
**Bone**	1.67 ± 0.94	0.41 ± 0.14	0.20 ± 0.19	0.48 ± 0.19	0.16 ± 0.02	0.04 ± 0.02
**HEK-SST2**	19.12 ± 4.47	28.41 ± 4.84	10.98 ± 4.69	14.94 ± 5.15	12.82 ± 3.09	3.82 ± 0.83
	**Tumor (T)-to-Non-Tumor Ratios**					
**T/Blood**	11	124	366	18	128	382
**T/Liver**	3.6	7.6	10	10	29	18
**T/Kidney**	0.4	1.1	3.2	1.6	2.3	3.3
**T/Muscle**	28	189	183	62	256	382
**T/Bone**	11	69	55	31	80	96

^a^ from [16] (own data, published as [^99m^Tc]Tc-TECANT-2).

**Table 3:** Biodistribution studies of [^99m^Tc]Tc-N4-JR11 and [^99m^Tc]Tc-HYNIC-Ahx-JR11 in SST2-expressing xenografts at 1 (*n* = 4), 4 (*n* = 4) and 24 (*n* = 3) hours p.i. Results are expressed as mean of the % injected activity per gram of tissue (% IA/g) ± standard deviation (SD).

Organ	[^99m^Tc]Tc-N4-JR11			[^99m^Tc]Tc-HYNIC-Ahx-JR11		
	1 h	4 h	24 h	1 h	4 h	24 h
**Blood**	0.84 ± 0.09	0.20 ± 0.02	0.02 ± 0.00	0.75 ± 0.12	0.12 ± 0.03	0.02 ± 0.00
**Heart**	0.57 ± 0.08	0.18 ± 0.01	0.04 ± 0.01	0.39 ± 0.05	0.09 ± 0.01	0.02 ± 0.00
**Lung**	8.99 ± 3.41	2.02 ± 0.87	0.27 ± 0.05	5.66 ± 0.68	0.58 ± 0.27	0.20 ± 0.08
**Liver**	1.79 ± 0.24	1.01 ± 0.13	0.45 ± 0.02	1.08 ± 0.15	0.70 ± 0.05	0.47 ± 0.06
**Pancreas**	29.14 ± 4.22	8.47 ± 1.92	0.60 ± 0.04	11.71 ± 2.62	1.64 ± 0.77	0.15 ± 0.02
**Spleen**	0.87 ± 0.09	0.33 ± 0.02	0.12 ± 0.01	1.87 ± 0.55	1.74 ± 0.39	1.52 ± 0.15
**Stomach**	29.32 ± 5.04	8.70 ± 0.80	1.12 ± 0.35	8.06 ± 0.84	1.51 ± 0.57	0.29 ± 0.03
**Intestine**	2.69 ± 0.94	3.21 ± 1.12	0.12 ± 0.03	1.78 ± 1.28	0.40 ± 0.23	0.16 ± 0.03
**Adrenal**	5.49 ± 1.22	2.36 ± 0.60	0.31 ± 0.02	1.05 ± 0.24	0.38 ± 0.05	0.19 ± 0.004
**Kidney**	12.76 ± 1.98	9.70 ± 0.85	2.23 ± 0.28	7.49 ± 1.21	5.26 ± 0.71	2.14 ± 0.11
**Muscle**	0.27 ± 0.05	0.09 ± 0.02	0.04 ± 0.01	0.21 ± 0.02	0.06 ± 0.03	0.03 ± 0.01
**Bone**	2.18 ± 0.43	0.32 ± 0.08	0.25 ± 0.02	0.58 ± 0.22	0.20 ± 0.05	0.14 ± 0.03
**HEK-SST2**	16.41 ± 2.87	19.07 ± 1.83	12.29 ± 1.59	14.22 ± 1.25	13.49 ± 2.07	5.83 ± 0.43
	**Tumor (T)-to-Non-Tumor Ratios**					
**T/Blood**	19	95	614	19	112	292
**T/Liver**	9.2	19	27	13	19	12
**T/Kidney**	1.3	2	5.5	1.9	2.6	2.7
**T/Muscle**	6.8	212	307	68	225	194
**T/Bone**	7.5	60	49	25	67	42

## Data Availability

The data presented in this study are available on request from the corresponding author.
